# Datasets for recognition of aggressive interactions of children toward robotic toys

**DOI:** 10.1016/j.dib.2020.106697

**Published:** 2020-12-26

**Authors:** Ahmad Yaser Alhaddad, John-John Cabibihan, Andrea Bonarini

**Affiliations:** a Department of Mechanical and Industrial Engineering, Qatar University, Doha 2713, Qatar; b Department of Electronics, Information and Bioengineering, Politecnico di Milano, Piazza Leonardo da Vinci 32, Milano 20133, Italy

**Keywords:** Social robots, Safety, Acceleration, Human-robot interaction

## Abstract

The data is related to unwanted interactions between a person and a small robotic toy based on acceleration sensor embedded within the robotic toy. Three toys were considered namely, a stuffed panda, a stuffed robot, and an excavator. Each toy was embedded with an accelerometer to record the interactions. Five different unwanted interactions were performed by adult participants and children. The considered interactions were hit, shake, throw, pickup, drop, and idle for the no interaction case. The collected data contains the magnitude of the resultant acceleration from the interactions. The data was processed by extracting the instances of interactions. A secondary dataset was created from the original one by creating artificial sequences. This data article contains the processed data that can be used to explore different machine learning models and techniques in classifying such interactions. Online repository contains the files: https://doi.org/10.7910/DVN/FHOO0Q.

**Specifications Table**

**Table:** 

Subject	Engineering
Specific subject area	Safety and Human-Robot Interaction
Type of data	Table (dataset)
How data were acquired	The data was acquired using a Sense Hat (i.e., add-on board) mounted on a raspberry pi. The Sense Hat contains an accelerometer that was used to acquire the resultant acceleration data. The device was embedded inside three robotic toys. Adult participants and children performed different unwanted interactions with the robotic toys. The data was then processed to extract the instances of interactions.
Data format	Raw Data
Parameters for data collection	The conditions for the data were the toys and the participants. Three different toys were considered. Adult participants and children took part in the experiments.
Description of data collection	Data was collected using an acquisition device based on raspberry Pi with an accelerometer embedded inside three robotic toys. Adult participants and children took part in performing the interactions with the three robotic toys.
Data source location	Institution: Qatar University
	City/Town/Region: Doha
	Country: Qatar
	Institution: Politecnico di Milano
	City/Town/Region: Milano
	Country: Italy
Data accessibility	Repository name: Harvard Dataverse
	Direct URL to data: https://doi.org/10.7910/DVN/FHOO0Q
Related research articles	Alhaddad, A.Y., Cabibihan, JJ. & Bonarini, A. Influence of Reaction Time in the Emotional Response of a Companion Robot to a Child's Aggressive Interaction. Int J of Soc Robotics (2020). https://doi.org/10.1007/s12369–020–00626-z

## Value of the Data

• The data characterize the unwanted physical interactions with small companion robots based on acceleration data.
• The data is useful to those working in the area of social robotics and the manufacturer of small robotic toys or companion robots.
• The data could be used to explore the efficacy of different machine learning techniques in characterizing interactions.
• The data can be exploited to promote safety in human-robot interaction.

## 1 Data Description

**Fig. 1:**
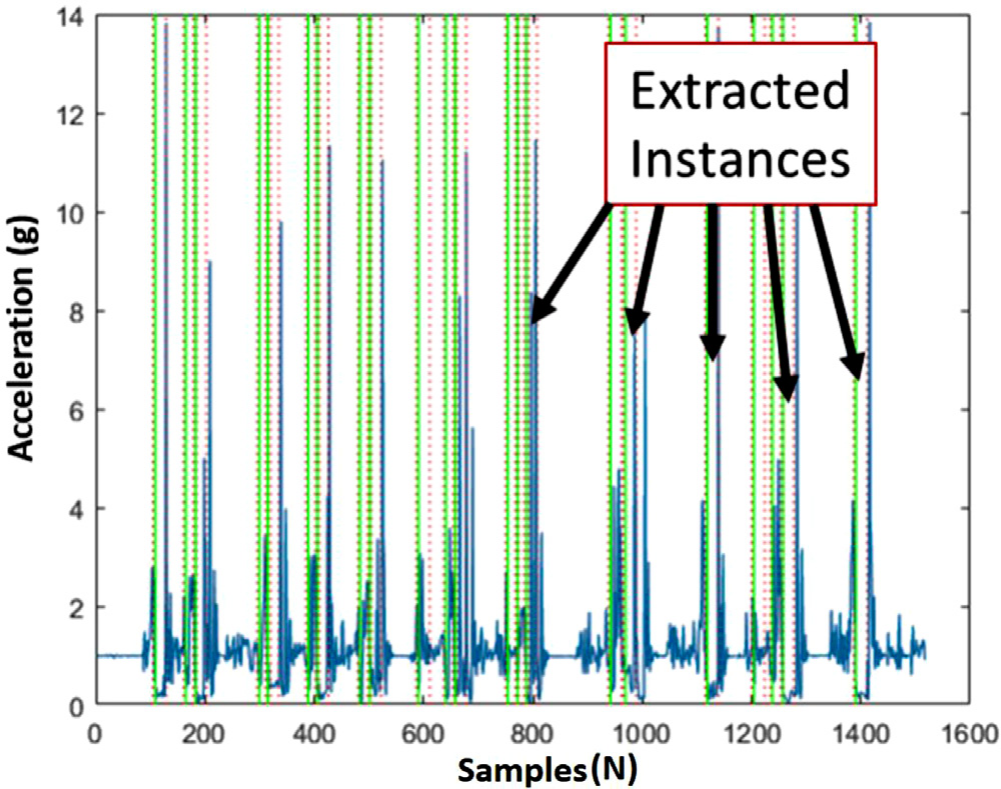
A sample of the extracted instances from the acceleration data for the throw behaviour (Reproduced from the related article [2]). The green lines show the start and the end of an instance. An instance consists of 25 acceleration data samples that corresponds to 0.86 s at 30 Hz acquisition rate.

**Fig. 2:**
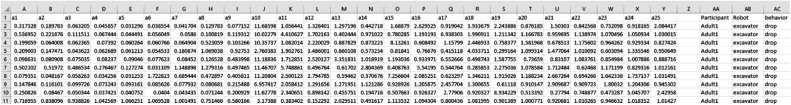
The arrangement of the data inside the dataset files. The first 25 columns (i.e., a1 till a25) represent the acceleration values. These values can be used as an input for a machine learning technique (e.g. neural network). The last three columns (i.e., AA, AB, and AC) indicate the participant number, the robotic toy used, and the behaviour. The behaviour column can be used as the output for the machine learning technique.

**Fig. 3:**
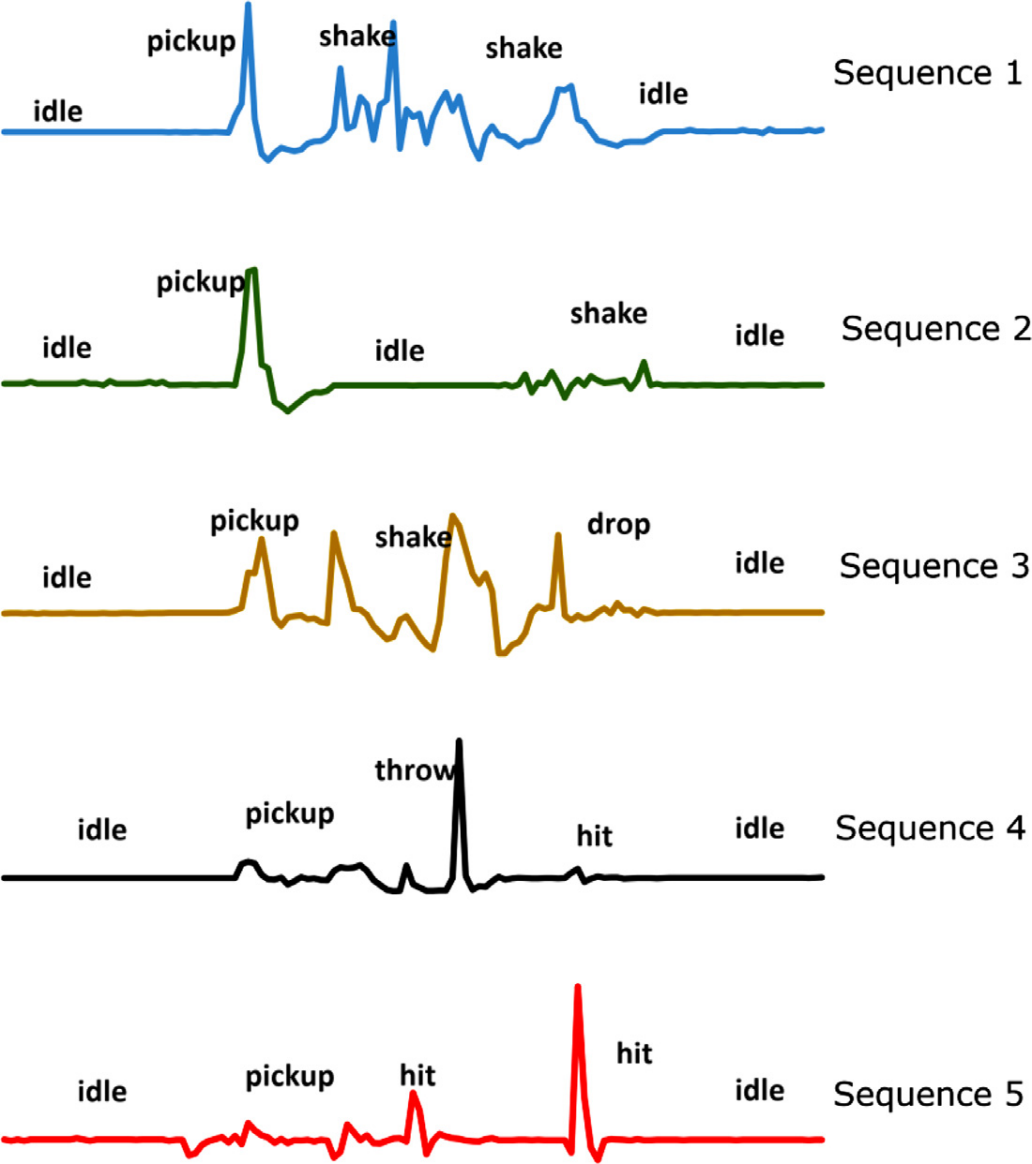
A sample of the artificial sequences that were created from the extracted instances (Reproduced from the related article [3]).

**Fig. 4:**
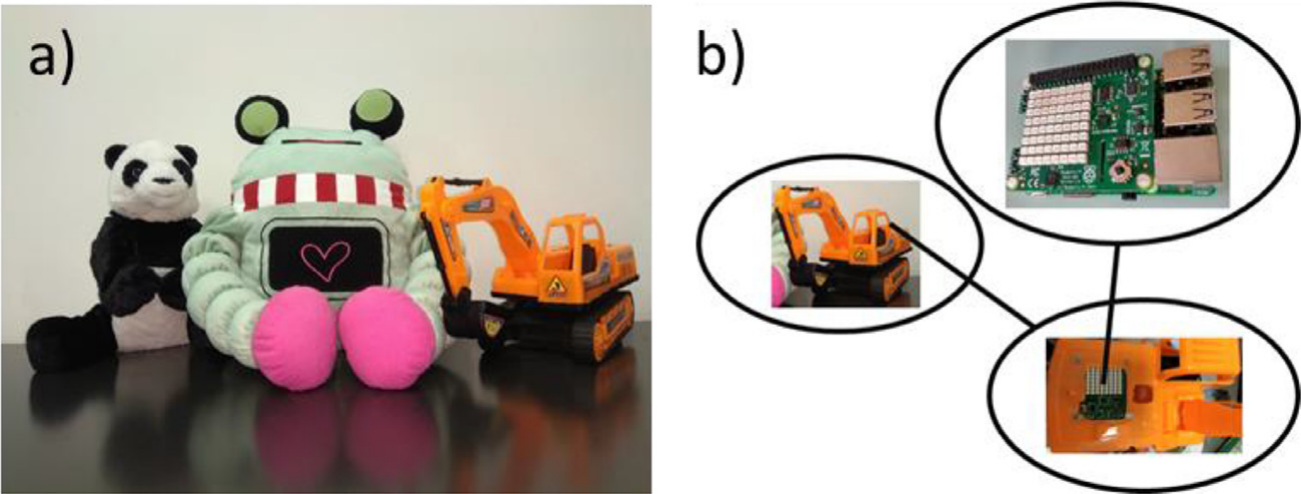
Overview of the experimental setup (Reproduced from the related article [2]). (a) The different toys that were considered. (b) The data acquisition device that was used to acquire the data.

The repository link contains six different datasets for the acceleration data [1]. The datasets are about participants (i.e., adults and children) performing different unwanted interactions with three different toys. For convenience, the datasets were subdivided to test and train based on the participants (e.g. adult and children). The first three datasets (i.e., *0_adult_test_dataset.csv, 0_adult_train_dataset.csv*, and *0_chidren_test_dataset.csv*) contains extracted instances of the behaviours of interest for the adult and children participants (Fig. 1). A behaviour instance consists of 25 data samples. Each file contains columns indicating the acceleration data, the participant number, the toy, and the behaviour (Fig. 2). A summary of the first three datasets is presented in Table 1. The second three datasets (i.e., *1_adult_seq_train_dataset.csv, 1_adult_seq_test_dataset.csv*, and *1_children_seq_test_dataset.csv*) contain artificial randomized sequences that were created from the first three datasets (Fig. 3). Three different toys were considered in the data collection process namely, a stuffed panda, a stuffed robot, and an excavator toy (Fig. 4). Further details about the data can be found in earlier work [2], [3].

**Table 1:** Summary of the first three datasets showing the number of extracted instances for each behaviour.

Dataset	Drop	Hit	Pickup	Shake	Throw	Idle
*0_adult_train_dataset.csv*	1000	1000	1000	1000	1000	1000
*0_adult_test_dataset.csv*	265	797	747	776	614	131
*0_chidren_test_dataset.csv*	49	195	56	377	70	0

## 2 Experimental Design, Materials and Methods

### 2.1 Experimental setup

Three toys were considered to collect the data. The toys were a stuffed robot, a stuffed panda, and an excavator (Fig. 4). The masses and the dimensions of the toys were in line with our earlier investigations pertaining safety in small robotic toys [4]. The range was selected because it enables ease of manipulation for children, which might pose as a source of harm during unwanted behaviours [4]. Each stuffed toy was modified by adding a zipper at the back to enable the ease of access to the inside. The toys were embedded with a data collection device and a battery (Fig. 4).

Raspberry Pi (Pi 3 Model B+, Raspberry Pi Foundation, UK) was considered as the data collection system. Raspbian operating system (V4.14, Raspberry Pi Foundation) was installed on a micro SD card (EVOplus, SAMSUNG). The size of the SD card was 32 GB and that should provide more than enough space for data, scripts, and operating system. TeamViewer (v13, US), a remote access software, was used to access the Rasberry Pi for debugging. All the components of the operating system (e.g. kernel, firmware, and packages) were updated to their recent versions. Sense Hat (Raspberry Pi Foundation, UK), which is an add-on board, was placed on top of the Raspberry pi. The Sense Hat contains different sensors to measure different modalities such as gyroscope, accelerometer, magnetometer, humidity, temperature, and barometric pressure.

The data collected mainly from the built-in accelerometer (LSM9DS1, STMicroelectronics, Switzerland) that was used to acquire the acceleration data at 30 Hz. This rate was reported to be enough to capture the characteristics of behaviours in similar applications [5]. The sensor collected the accelerations data in the X, Y, and Z direction. The resultant magnitude acceleration was calculated from the individual accelerations and it was considered in producing the datasets. A simple Python script was used to read the accelerometer readings, perform the calculation of resultant acceleration, and then store the values in (CSV) files.

### 2.2 Procedures

Six adults (aged 24 to 31 years old) and four children (aged 4 to 9 years old) participated in acquiring the data. For the adult sessions, each participant was asked to perform five different unwanted interactions with each robotic toy at different times or days for a certain duration (i.e., around 5 min for each behaviour). The interactions were hit, shake, throw, pickup, and drop. The idle case was acquired separately for each toy. Freedom was given to the participants in the way the interactions were performed. The interactions of one participant with each toy generated a varying number of instances. However, the total instances considered from one participant for one behaviour (e.g. hit) included all interactions with the three toys. These instances from each participant were merged and made into training and testing datasets. The contribution of each participant in the datasets were balanced based on the total acquire instances from their individual interactions. As for the children, imaginative scenarios were told to acquire the interactions [2], [3]. For example, the robot is sleeping and you need to shake it to wake it up.

The instances of interactions were extracted from the collected data using a MATLAB script (MathWorks, Massachusetts, USA) based on certain thresholds (Fig. 1) [2]. Each behaviour has certain characteristics that distinguishes them from others. The thresholds considered were based on these characteristics. For example, a throw behaviour is characterized by a small spike followed by a drop in the acceleration and finally a large spike. Based on the selected threshold, the script recognizes samples of interest. Then these samples were manually inspected for accuracy and correctness. The selected size for the extracted instances was set to 25 data samples. As for the artificial sequences, a Python script was used to generate sequences with randomized instances from the extracted data. The sequences were selected based on their likelihood of occurrence in a realistic scenario. More details about the participants, experimental setup, and procedures can be found in earlier work [2], [3].

## Ethics Statement

An informed consent was obtained from each participant or his/her guardian. The procedures did not include invasive or potentially dangerous methods and were in accordance with the Code of Ethics of the World Medical Association (Declaration of Helsinki).

## CRediT Author Statement

**Ahmad Yaser Alhaddad:** Data curation, Hardware, Software, Writing- Original draft preparation. **John-John Cabibihan:** Conceptualization, Funding, Reviewing. **Andrea:** Methodology, Reviewing, Editing.

## Declaration of Competing Interest

The authors declare that they have no known competing financial interests or personal relationships which have, or could be perceived to have, influenced the work reported in this article.
